# AQUACEL® Ag Advantage reduces the stress of postoperative packing removal after endoscopic sinus surgery

**DOI:** 10.1016/j.bjorl.2023.101292

**Published:** 2023-07-18

**Authors:** Kei Hosoya, Yohei Maeda, Taro Komachi, Kazuki Sato, Kimihiro Okubo

**Affiliations:** a Musashi Kosugi Hospital, Nippon Medical School, Department of Otolaryngology, Nakahara-Ku, Kawasaki, Japan; b Tama Nagayama Hospital, Nippon Medical School, Department of Otolaryngology, Tama, Tokyo, Japan; c Japan Community Health Care Organization Osaka Hospital, Department of Otorhinolaryngology, Fukushima, Osaka, Japan; d Osaka University, Department of Otorhinolaryngology-Head and Neck Surgery, Suita, Japan; e Chiba Hokusoh Hospital, Nippon Medical School, Department of Otolaryngology, Inzai, Japan; f Nippon Medical School Hospital, Department of Otolaryngology, Head and Neck Surgery, Bunkyo-Ku, Tokyo, Japan

**Keywords:** Endoscopic sinus surgery, Nasal packing, AQUACEL® Ag advantage, Stress

## Abstract

**Objective:**

After Endoscopic Sinus Surgery (ESS), packing plays an important role in wound healing and hemostasis. However, the effect of the packing removal procedure on physician stress has not been evaluated. The purpose of this study was to evaluate physician stress during packing removal for patients treated with AQUACEL® Ag Advantage versus KALTOSTAT®.

**Methods:**

This retrospective study included 15 patients who underwent packing with ESS for chronic rhinosinusitis performed at two centers; 9 were treated with AQUACEL® Ag Advantage and 6 were treated with KALTOSTAT®. Physician stress during packing removal was evaluated with the National Aeronautics and Space Administration-Task Load Index (NASA-TLX). The time required to remove the packing and the number of instruments used in the procedure were recorded. Postoperative bleeding (Boezaart bleeding score) and wound healing were graded. Patient symptoms on the day after surgery and pain during packing removal were assessed using a visual analog scale.

**Results:**

Computed tomography scores, asthma complications, and blood eosinophil counts were significantly higher in the AQUACEL® Ag Advantage group. Patient symptoms on the day after surgery were not significantly different between the two groups. Physician stress during the task of packing removal was significantly lower in the AQUACEL® Ag Advantage group than in the KALTOSTAT® group (35.5 vs. 81.0, *p* = 0.016) according to the NASA-TLX scores. The number of instruments used in the procedure was significantly lower in the AQUACEL® Ag Advantage group than in the KALTOSTAT® group (3.0 vs. 6.0, *p* = 0.015). There were no significant differences in procedure time for packing removal, postoperative bleeding, wound healing, or patient pain at the time of packing removal between the groups.

**Conclusion:**

Physicians feel stressed about packing removal. In addition, AQUACEL® Ag Advantage is useful for packing after ESS, requiring fewer instruments for the procedure than KALTOSTAT® and reducing physician stress about the procedure.

**Level of evidence:**

Level 3.

## Highlights

• Packing removal after endoscopic nasal sinus surgery causes stress to the physician.
• Physician stress in packing removal is lower with AQUACEL® Ag Advantage than control.
• AQUACEL® Ag Advantage is useful for packing after endoscopic nasal sinus surgery.

## Introduction

Endoscopic Sinus Surgery (ESS) is the standard treatment for chronic rhinosinusitis. It is a procedure performed to decrease symptoms. Compared to traditional surgery that involves external incisions, ESS is less invasive and offers significant benefits to patients. However, 86.9% of surgeons who perform minimally invasive surgery report physical symptoms and discomfort, indicating that ESS is associated with a high workload.^1^ In particular, 77% of otolaryngologists experience musculoskeletal symptoms with ESS and suffer from physical discomfort and symptoms resulting from ESS.^2^

Ramakrishnan and Milam evaluated the physical fatigue load of surgeons performing ESS using the National Aeronautics and Space Administration Task Load Index Survey (NASA-TLX).^3^ The NASA-TLX has been used to assess potential changes in performance in a variety of areas, including workload for operating cars and airplanes. More recently, it has been used for assessing workload and physical fatigue in surgery.^4^, ^5^ The NASA-TLX is a subjective workload physical fatigue assessment scale consisting of six items: mental demand, physical demand, temporal demand, performance, effort, and frustration. The NASA-TLX has shown that ESS is a highly stressful surgery,^6^ revealing physical damage to the ESS surgeon.

Packing is often used after endoscopic nasal sinus surgery for hemostasis and wound healing.^7^, ^8^ Various types of packing have been investigated for their hemostatic and wound-healing effects. AQUACEL® Ag is a hydrofiber wound dressing made of carboxymethylcellulose that contains silver with antimicrobial properties.^9^ It is a packing material that is commonly used in Japan. KALTOSTAT® is a wound dressing composed of calcium sodium alginate that promotes hemostasis by exchanging calcium for sodium ions and has been reportedly used as packing for ESS.^10^ It absorbs the exudate via capillary action and forms a gel to promote wound healing.^11^

Although physician stress during ESS is becoming increasingly recognized, physician stress during packing removal has not yet been clarified. In this study, physician stress was evaluated when post-ESS packing with AQUACEL® Ag Advantage or KALTOSTAT® was removed. In addition, we evaluated whether AQUACEL® Ag Advantage, which is sutured in place, causes less stress to the physician at the time of removal than KALTOSTAT®, which becomes brittle after gelation.

## Methods

This retrospective cohort study included 15 patients who underwent bilateral ESS under general anesthesia for chronic rhinosinusitis from January 1 to March 11, 2021 at Nippon Medical School Tama Nagayama Hospital and Nippon Medical School Chiba Hokusoh Hospital and postoperative removal of AQUACEL® Ag Advantage (Convatec Japan, Tokyo, Japan) or KALTOSTAT® (Convatec Japan, Tokyo, Japan). Exclusion criteria included: reoperation, patient age under 20 years, packing with other materials, and history of systemic diseases such as Kartagener’s syndrome. Each hospital has one surgeon with extensive ESS experience. Septoplasty and submucosal inferior turbinectomy were performed as needed.

We collected data on the following characteristics via medical records: gender, age, nasal polyp score, Computed Tomography (CT) score (Lundy–Mackay staging system), presence of Eosinophilic Chronic Rhinosinusitis (ECRS), asthma, allergic rhinitis, peripheral eosinophil count, total IgE, and additional surgeries.

**Figure 1:**
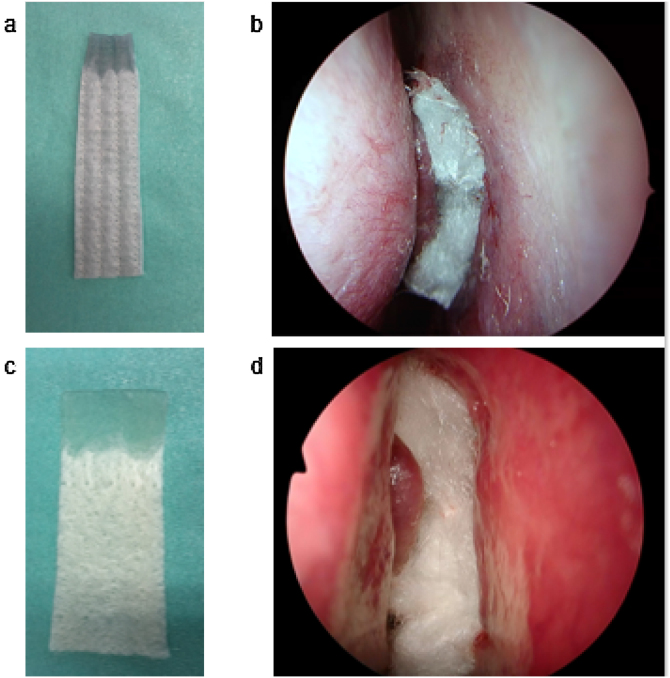
(a) AQUACEL® Ag Advantage cut into quarters. The upper part was gelatinized with water. (b) AQUACEL® Ag Advantage was inserted as nasal packing after endoscopic sinus surgery. (c) KALTOSTAT® cut into quarters. The upper part was gelatinized with water. (d) KALTOSTAT® was inserted as nasal packing after endoscopic sinus surgery.

AQUACEL® Ag Advantage (10 × 10 cm) packing material was divided into four sections along the stitches. A total of two sheets were placed in the bilateral middle and common nasal passages (Fig. 1). KALTOSTAT® (7.5 × 12 cm) packing material was divided into two sections. A total of two sheets or less was placed in the bilateral middle and common nasal passages. Packing placed in the common nasal passages was removed on Postoperative Day (POD) 2. Nasal irrigation was started on POD 3–6.

The antibiotic cefazolin (2 g) was infused on the day of surgery. Oral cefditoren pivoxil (300 mg/day) was used for 5 days after surgery. Acetaminophen (1,000 mg) infusion was used for pain control on the day of surgery if requested by the patient.

On POD-1, the patient was given a document to record their Visual Analog Scale (VAS) score (0–100) for nasal pain, headache, rhinorrhea, and posterior rhinorrhea. The surgeon removed the packing and cleaned the nasal lining. The date of removal, time required for removal, and number of instruments used were recorded. The patient was given a document to describe the pain of the packing removal procedure with a VAS score. The condition of the nose was checked endoscopically, and bleeding was evaluated using the Boezaart score.^12^ Mucosal edema (0–3), infection (0–2), crusting (0–2), granulations (0–3), and adhesions (0–3) were also graded.^13^ Bleeding score and sinus findings were evaluated by two surgeons based on anonymized photographs. The average score of the left and right sides was calculated. After the physician performed the packing removal procedure, physician stress was assessed with a VAS using the NASA-TLX.

This study was implemented in accordance with the Declaration of Helsinki. It was reviewed and approved by the ethics committee of our institution (approval number: blinded for review). Information about the purpose and implementation of the study was posted on the homepage of our institution.

### Statistical analysis

Data are presented as medians and IQR (interquartile range); the Mann–Whitney *U* test and Fisher’s exact test were used for comparisons between the two groups. A two-tailed *p* < 0.05 was considered statistically significant.

## Results

Table 1 shows the background characteristics of the study patients in the AQUACEL® Ag Advantage and KALTOSTAT® groups. At Nippon Medical School Tama Nagayama Hospital, 4 patients received AQUACEL® Ag Advantage and 3 patients received KALTOSTAT®. At Nippon Medical School Chiba Hokusoh Hospital, 5 patients received AQUACEL® Ag Advantage and 3 patients received KALTOSTAT®. CT scores were significantly higher in the AQUACEL® Ag Advantage group than in the KALTOSTAT® group (19.0 vs. 12.0, *p* = 0.014). The prevalence of ECRS was 88.9% (8 patients) in the AQUACEL® Ag Advantage group and 66.7% (4 patients) in the KALTOSTAT® group. Asthma complications (5 vs. 0 patients, *p* = 0.044) and blood eosinophil count (5.8% vs. 2.4%, *p* = 0.020) were significantly higher in the AQUACEL® Ag Advantage group compared with the KALTOSTAT® group.

**Table 1:** Clinical characteristics of the study patients.

	AQUACEL® Ag Advantage group (n = 9)	KALTOSTAT® group (n = 6)	*p*-value
Gender, female: male (n)	8:1	5:1	1
Age, years, median (IQR)	46.0 (37.0–53.0)	60.5 (52.5–67.8)	0.189
Total polyp score, median, (IQR)	6.0 (6.0)	4.0 (2.3–5.8)	0.089
CT score, median, (IQR)	19.0 (16.0–20.0)	12.0 (9.5–13.8)	0.014^a^
ECRS, n (%)	8 (88.9)	4 (66.7)	0.525
Asthma, n (%)	5 (55.6)	0 (0)	0.044^a^
Allergic rhinitis, n (%)	7 (77.8)	3 (50.0)	0.328
Peripheral eosinophil count (%), median (IQR)	5.8 (5.2–7.2)	2.4 (1.6–4.0)	0.020^a^
Total IgE, IU/mL, median (IQR)	303.0 (114.0–456.0)	58.0 (37.5–300.5)	0.114
Additional surgery, n (%)	9 (100.0)	6 (100.0)	1
Sep + SIT (n)	3	3	
Sep (n)	6	3	

Data are expressed as medians (IQR).

IQR, Interquartile Range; CT, Computed Tomography; ECRS, Eosinophilic Chronic Rhinosinusitis; Sep, Septorhinoplasty; SIT, Submucosal Inferior Turbinectomy.

a Denote statistical significance at *p* < 0.05 with the Mann–Whitney *U* test or Fisher’s exact test.

Physician stress during the packing removal procedure was assessed using the NASA-TLX (Table 2). Overall, mental demand 51.0 (28.0–71.0), physical demand 32.0 (28.5–70.5), and temporal demand 43.0 (29.5–85.0) were somewhat high. Performance, which indicates the degree of satisfaction with the work in terms of goal attainment, was very high in both groups (80.0 vs. 90.0, *p* = 0.977). The median VAS score for the AQUACEL® Ag Advantage group was 35.5, which was lower than that of the KALTOSTAT® group (*p* = 0.016). Regarding frustration, the overall score was medium in the AQUACEL® Ag Advantage and KALTOSTAT® groups, respectively (12.5 vs. 38.5, *p* = 0.106).

**Table 2:** NASA-TLX (VAS 0–100) results from physicians regarding packing removal.

	AQUACEL® Ag Advantage group	KALTOSTAT® group	Overall	*p*-value
Mental demand	31.0 (20.8–55.8)	63.9 (54.4–84.0)	51.0 (28.0–71.0)	0.136
Physical demand	30.0 (21.0–41.3)	62.5 (44.2–87.0)	32.0 (28.5–70.5)	0.112
Temporal demand	32.0 (18.8–47.3)	74.5 (50.1–93.0)	43.0 (29.5–85.0)	0.119
Performance	80.0 (52.5–93.0)	90.0 (83.0–92.5)	90.0 (76.0–92.0)	0.977
Effort	35.5 (22.3–50.3)	81.0 (55.2–95.8)	50.0 (41.5–80.0)	0.016^a^
Frustration	12.5 (8.8–24.3)	38.5 (24.9–58.3)	27.0 (10.0–56.0)	0.106

Data are expressed as medians (IQR).

NASA-TLX, National Aeronautics and Space Administration-Task Load Index; VAS, Visual Analog Scale; IQR, Interquartile range.

a Denote statistical significance at *p* < 0.05 with the Mann–Whitney *U* test.

Table 3 summarizes the evaluation of the procedure on the day the packing was removed; the AQUACEL® Ag Advantage group had packing removed at a median of 11.0 days after surgery and the KALTOSTAT® group underwent removal at a median of 10.0 days after surgery (*p* = 0.574). Patient pain and procedure time were not significantly different, but the number of instruments required for the procedure was significantly lower in the AQUACEL® Ag Advantage group than in the KALTOSTAT® group (3.0 vs. 6.0, *p* = 0.015).

**Table 3:** Timing of nasal packing removal, patient pain score (VAS 0–100), procedure time, and number of instruments used in nasal packing removal.

	AQUACEL® Ag Advantage group	KALTOSTAT® group	*p*-value
Postoperative day of nasal packing removal	11.0 (8.0–13.0)	10.0 (6.8–11.8)	0.574
Pain during procedure	4.0 (2.0–7.1)	1.8 (1.4–2.0)	0.313
Procedure time (minutes)	18.4 (18.0–26.0)	30.5 (25.3–35.7)	0.181
Number of instruments	3.0 (3.0–4.0)	6.0 (5.3–6.8)	0.015^a^

Data are expressed as medians (IQR).

VAS, Visual Analog Scale; IQR, Interquartile Range.

a Denote statistically significant at *p* < 0.05 by Mann–Whitney *U* test.

Table 4 compares the mean Boezaart score, mucosal edema, infection, crusting, granulation, and adhesion scores in the AQUACEL® Ag Advantage and KALTOSTAT® groups at the time of packing removal. There were no significant differences in intranasal findings between the two groups.

**Table 4:** Boezaart scores and wound healing scores.

	AQUACEL® Ag Advantage group	KALTOSTAT® group	*p*-value
Boezaart score	1.0 (1.0)	1.0 (1.0)	0.802
Mucosal edema	1.0 (1.0)	1.0 (1.0)	0.618
Pus	0.5 (0–1.0)	0	0.062
Crusting	0	0	>0.999
Granulation	0 (0–1.0)	0	0.327
Adhesion	0	0	>0.999

Data are expressed as medians (IQR).

IQR, Interquartile Range.

Table 5 compares patients’ subjective symptoms of nasal pain, headache, rhinorrhea, and posterior rhinorrhea on the day after surgery using VAS scores by group. No significant differences were found between the two groups.

**Table 5:** Symptom scores (VAS 0–100) reported by patients on the day after surgery.

	AQUACEL® Ag Advantage group	KALTOSTAT® group	*p*-value
Nasal pain	9.0 (4.0–36.0)	2.5 (0.5–9.0)	0.340
Headache	35.0 (2.0–46.0)	4.0 (0–18.5)	0.173
Nasal bleeding	8.0 (2.0–41.0)	24.0 (13.5–44.3)	0.507
Postnasal drip	26.0 (3.0–52.0)	14.5 (6.0–34.3)	0.626

Data are expressed as medians (IQR).

IQR, Interquartile Range.

## Discussion

Removal of ESS packing proved to be stressful for the physician. Postoperative packing from ESS with AQUACEL® Ag Advantage caused less stress to the physician during removal and required fewer instruments. Postoperative patient pain and bleeding were comparable for AQUACEL® Ag Advantage and KALTOSTAT®. Bleeding at the time of packing removal and wound healing were also comparable between AQUACEL® Ag Advantage and KALTOSTAT®. CT scores, asthma complications, and blood eosinophil counts were significantly higher in the AQUACEL® Ag Advantage group. Despite the higher number of severe cases in the AQUACEL® Ag Advantage group, there was less stress experienced by the physician from packing removal.

ESS has recently been reported to be taxing on the surgeon’s body (2, 3, 14), with 77% of otolaryngologists having experienced musculoskeletal symptoms^2^ and 60% having experienced back and neck pain.^14^ They reported discomfort in the feet, lower back, upper back, and arm after ESS.^3^ The NASA-TLX score was approximately 40 overall,^3^ indicating that the surgeon was under stress when performing surgery in the standing or sitting position. However, there has been no validation of physician stress during the packing removal procedure after ESS.

The NASA-TLX results for AQUACEL® Ag Advantage and KALTOSTAT®, which are insoluble and require extraction, showed that physicians reported stress during extraction (Table 2). When AQUACEL® Ag Advantage is packed in the sinuses, it becomes gelatinized with saline solution from intranasal exudate and nasal gargling. Gelatinized AQUACEL® Ag Advantage remained in the sinus cavity as a mass because of the sutures and the physician was able to remove it smoothly with nasal forceps, Nishihata’s small cup-shaped nasal forceps straight forward, and angled suction cannulas. On the other hand, KALTOSTAT® in the sinus cavity becomes gelatinized as well, but it is fragile and collapses because it is not sutured. Moreover, KALTOSTAT® in the sinus cavity could migrate into the maxillary sinus after nasal irrigation. Therefore, it was difficult to grasp and extract KALTOSTAT®; the effort required was considered to be very high. In addition to the instruments used to remove AQUACEL® Ag Advantage, Heymann forceps and Nishihata’s small cup-shaped nasal forceps curved up forward were needed, increasing the number of instruments used (Table 3). There were no significant differences in the date of packing removal, patient pain during the procedure, or time required for the procedure. Performance measured with the NASA-TLX, which indicates physician satisfaction with their work, was very high in both groups, suggesting that although the KALTOSTAT® group had a more difficult packing removal procedure, both products had similar reliability. The KALTOSTAT® group was more difficult to treat, but the postoperative treatment was performed as reliably as in the AQUACEL® Ag Advantage group.

No differences were observed between the two groups in terms of intranasal bleeding or wound healing when the packing material was removed (Table 4). No significant differences were found between the two groups in terms of patient pain or blood loss on the day after surgery (Table 5). It has been reported that long-term implantation promotes wound healing.^15^ In the present study, the packing was in place for approximately 10 days, which was thought to promote wound healing, resulting in lower wound healing scores in both groups. The reason for less blood loss was thought to be that wound healing had progressed and the gelatinized packing material did not adhere to the wound, resulting in less tissue damage at the time of removal. Although many types of packing materials are used in ESS, the stress on the surgeon has not been investigated. We hope that in the future, when considering the performance of packing materials, attention will be paid to the stress on the surgeon.

### Limitations

This study had a few limitations. The sample size was small and there were differences in background characteristics of the two groups. Because this was not a blinded study, there could have been physician bias in terms of stress and time spent on the procedure.

## Conclusion

Packing removal after ESS was found to be stressful for physicians. When packing with AQUACEL® Ag Advantage and KALTOSTAT® was compared, the number of instruments used for removal and the stress of the procedure on the physician were significantly lower with AQUACEL® Ag Advantage. The results suggest that AQUACEL® Ag Advantage might reduce physician stress related to packing removal after ESS more than KALTOSTAT®.

## Funding

This work was supported JSPS KAKENHI grant number JP20K0971 (to YM).

## Conflicts of interest

The authors declare no conflicts of interest.
